# Prevalence and Pattern of Birth Defects in Saudi Arabia: A Systematic Review of Observational Studies

**DOI:** 10.3390/pediatric15030040

**Published:** 2023-07-20

**Authors:** Ebtihal Elameen Eltyeb, Majidah Hussain Asiri Halawi, Thekra Badr Mohammed Tashari, Khaled Alharbi, Ohoud Saad Alsayari, Danah Abdullah Albarrak, Raga Abdelkhalig Eltayeb, Ali Ali Ahmed Al-Makramani, Isameldin Elamin M. Medani

**Affiliations:** 1 Faculty of Medicine, Jazan University, Jizan 45142, Saudi Arabia; halawi.majidah@gmail.com (M.H.A.H.); tkekra1999@gmail.com (T.B.M.T.); reltayeb@jazanu.edu.sa (R.A.E.); makra3@yahoo.com (A.A.A.A.-M.); isameldin2015@gmail.com (I.E.M.M.); 2 Imam Abdulrahman Alfaisal Hospital, Riyadh 14723, Saudi Arabia; dr.alharbikhaled@gmail.com; 3 College of Medicine, King Saud bin Abdulaziz University, Riyadh 14611, Saudi Arabiadana.albarrak0@gmail.com (D.A.A.)

**Keywords:** birth defects, pattern, prevalence, Saudi Arabia

## Abstract

Introduction: Birth defects are a significant concern since they can lead to permanent disability and death. This study comprehensively reviews the prevalence and patterns of birth defects in Saudi Arabia. Methods: A systematic analysis of the literature retrieved from three databases (Pub Med, Science Direct, and the Saudi digital library) published between 1989 and 2022 was performed. Observational studies that addressed the prevalence and patterns of birth defects in Saudi Arabia were chosen based on the eligibility criteria, while systematic reviews, review articles, non-relevant articles, and studies that did not fulfill the eligibility criteria were excluded. Quality and risk of bias were evaluated based on the JBI and GRADE tools, respectively. Results: We identified 26 eligible publications of 1277 records that included 297,668 patients from different regions of Saudi Arabia. The highest overall prevalence of birth defects was 46.5 per 1000 live births compared to a lowest rate of 8.6 per 1000 in one study. Several studies have reported positive associations of consanguinity, maternal folic acid supplementation, family history of birth defects or genetic abnormalities, and maternal co-morbidities. The most frequent birth defects include cardiac, genitourinary, craniofacial, and nervous system defects. Conclusion: Robust findings have improved our understanding of the prevalence and pattern of birth defects in Saudi Arabia. Importantly, future studies will likely require multicenter collaboration to arrive at appropriate sample sizes in the context of the effects of risk factors on elevated prevalence. Furthermore, quantitative data require careful evaluation in more complex statistical models.

## 1. Introduction

Birth defects (BDs), congenital anomalies, or congenital malformations develop during intrauterine life and may or may not be discovered until later in life. These defects result in lifelong disability and mortality, contributing to almost 20% of infant deaths [1,2]. The World Health Organization (WHO) described a BD as a significant abnormality that presents as physical, functional, or mental disability at birth [3]. Birth defects may impact one or more organ systems and might be isolated or appear in a recognizable combination or pattern. The four main recognized patterns of BDs are syndromes, sequences, developmental field defects, and associations. A syndrome refers to a particular BD pattern associated with unique clinical features. A sequence occurs when a single malformation results in the development of subsequent anomalies. At the same time, developmental field defects defined as a pattern of BDs caused by an underlying disturbance in a localized embryological region. Last, an association refers to a pattern of malformations that do not share an exact pathogenesis but occur more frequently together than by chance [4,5].

Birth defects occur at varying frequency worldwide, and according to a global report, 3.3 million children younger than the age of 5 die from significant birth abnormalities every year. [6]. The prevalence in the United States was 29.2 per 1000 live births; in Europe it was estimated by the European Surveillance of Congenital Anomalies (EUROCAT)to be 23.9 per 1000, while in sub-Saharan Africa, it is 20.4 per 1000 live births [7,8,9].

Although most BDs are idiopathic, 10% of BDs arise from modifiable risks that can be changed or revised, and primary birth BD prevention consists of promoting healthy eating habits and preventing maternal infections during pregnancy. These modifiable risks include maternal medical conditions, substance abuse, infection, medications, radiation, hyperthermia, and folic acid insufficiency [10,11,12]. However, non-modifiable risk factors, such as maternal age, family history, and a previously affected child, cannot be changed or prevented, thus influencing BD development [13,14].

In Saudi Arabia, birth defects remain the leading cause of death among children, with high rates of consanguineous marriage and genetic diseases [15,16]. Many independent studies have been conducted in Saudi Arabia, and a significant variation in the prevalence of birth defects has been observed. The absence of public health measures, especially in high-risk populations, is often attributed to the lack of epidemiological data. This systematic review aimed to assess the prevalence of birth defects in Saudi Arabia and determine the patterns according to the available data.

## 2. Methods

### 2.1. Study Design and Registration

This systematic review aimed to estimate the prevalence and pattern of birth defects in Saudi Arabia. In addition, similarities and disparities in the existing evidence and literature were examined to obtain conclusive results. The International Prospective Registry of Systematic Reviews (PROSPERO) registered this systematic review protocol on 25 February 2023 under registration number CRD42023398821.

### 2.2. Search Strategy

We conducted electronic literature searches, including of Pub Med (National Library of Medicine), Science Direct, and the Saudi digital library, for studies published between January 1989 and January 2022. A structured design based on the Preferred Reporting Items for Systematic Review and Meta-Analyses (PRISMA) Guidelines and a checklist were used to select and review studies included in the review, and they were used as a foundation for reporting systematic reviews with goals other than evaluating interventions [17]. More details about PRISMA checklists are provided in Table S1 in the Supplementary Document.

In addition, we conducted Medical Subjects Heading (MeSH Database) and keyword searches for non-MeSH data. The keywords employed for the search were “Prevalence” OR “Epidemiology” AND “Birth defects” OR “Congenital Abnormalities” OR “Congenital Malformation” OR “Congenital Anomalies” AND “Pattern” OR “types” OR “sub-types” AND “Saudi Arabia” OR “KSA”. A manual search for identified references from included studies, relevant reviews, and gray literature was performed to find further relevant studies not found in the database search.

### 2.3. Study Selection and Eligibility Criteria

This review included original observational research that included cohort, case-control, cross-sectional studies, case reports, and case series published in English, as no related studies in Arabic were found within the aforementioned time. In addition, systematic reviews, review articles, non-relevant articles, and studies that did not fulfill the eligibility criteria were excluded. Two researchers (TT and MH) screened the studies and assessed their eligibility for inclusion. Data abstraction was mediated by three researchers (KA, EE, and OA). Subsequently, one researcher (EE) assessed the quality of each study.

PICO was used to define the inclusion criteria as follows.

Population/Patients: Patients (males and females of all ages) diagnosed with birth defects, including cardiac, craniofacial, musculoskeletal, nervous, genitourinary, gastroenterological, and chromosomal defects. Study eligibility criteria: Inclusion Criteria: Patients diagnosed with birth defects, studies that calculated the prevalence of birth defects, studies that calculated risk factors of birth defects, and studies reporting the characteristics of different patterns of birth defects. The exclusion criteria omitted studies involving non-humans, opinion papers, editorials, previous reviews, studies validating epidemiological methods, and non-accessible articles.

Intervention/Exposure: This review summarizes data on the epidemiology of birth defects, specifically the prevalence.

Control: Not Applicable.

Outcomes: The primary outcomes of this systematic review were the prevalence and patterns of birth defects in Saudi Arabia. The number of newborns delivered with birth defects divided by the total number of babies born during the study period who were enrolled in the study multiplied by 1000 was calculated to estimate the prevalence. At the same time, the main group patterns of birth defects were broadly classified according to the International Classification of Diseases coding system into the nervous, cardiovascular, genitourinary, craniofacial, musculoskeletal, and gastrointestinal systems and chromosomal defects [18]. In addition, subgroups were considered, as some studies reported specific patterns alone, such as cleft lip and palate or congenital heart disease. Other outcomes, such as the risk factors and clinical outcomes also were also considered.

### 2.4. Data Extraction and Management

Two authors screened the titles and abstracts, and then all authors discussed the full texts to apply the inclusion criteria and to discuss any disagreement regarding study selection. Studies that did not meet the eligibility criteria were documented for reasons and excluded. Data were extracted manually and transferred to data extraction forms and included the following: name of the first author, year of publication, location, study design, total number of participants, and sample size of individual studies, including the number of patients who were diagnosed with a birth defect, characteristics of the participants (age, gender, associated co-morbidities), methods of diagnosis, and pattern of birth defect.

### 2.5. Synthesis of the Evidence

**Figure 1:**
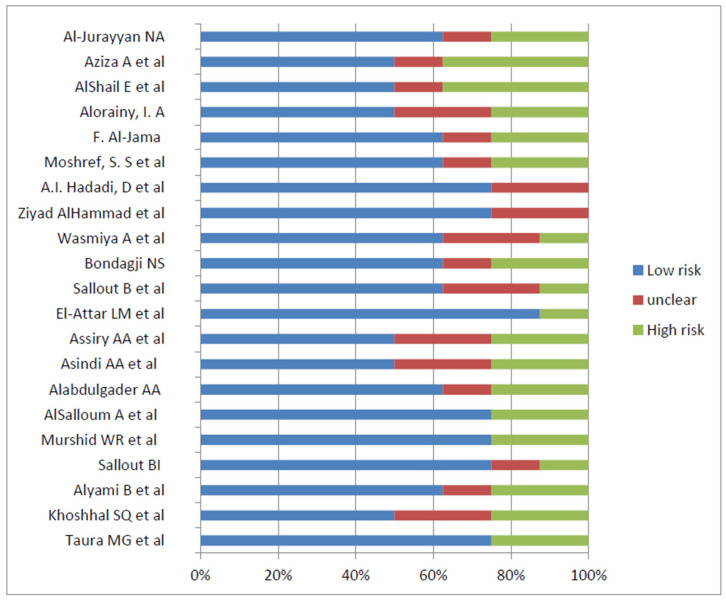
Risk of bias of cross sectional studies utilizing JBI checklist [20,21,22,23,24,25,26,27,28,29,30,31,32,33,34,35,36,37,38,39,40].

**Figure 2:**
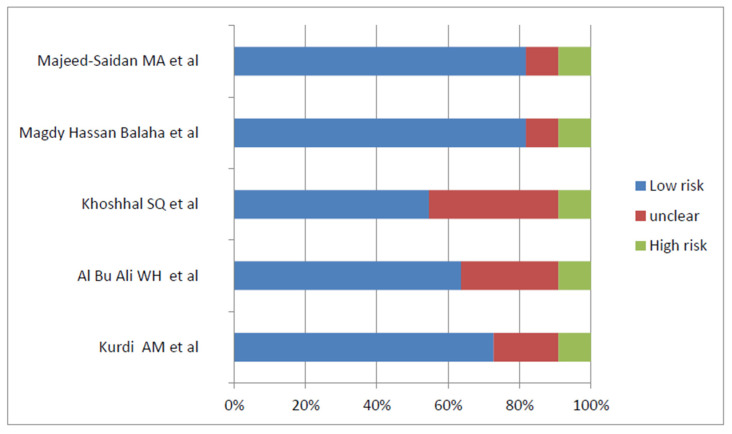
Risk of bias of cohort studies utilizing JBI checklist [21,41,42,43,44].

The *Joanna Briggs Institute* (JBI) provides freely available critical assessment tools for systematic reviews, designed to be study-specific and presented as checklist questions. A critical assessment checklist for observational studies is used to evaluate the quality, reliability, validity, and relevance to practice of the studies [19]. Two JBI checklists were used: one for cohort studies and the other for cross-sectional studies, as shown in the bar charts in Figure 1 and Figure 2. For certainty of evidence, the GRADE working group graded the evidence used and rated it as high, moderate, low, or very low certainty (GRADE). Subgroup and stratified analyses were performed according to age, sex, and birth defect patterns.

## 3. Results

### 3.1. Search Results

**Figure 3:**
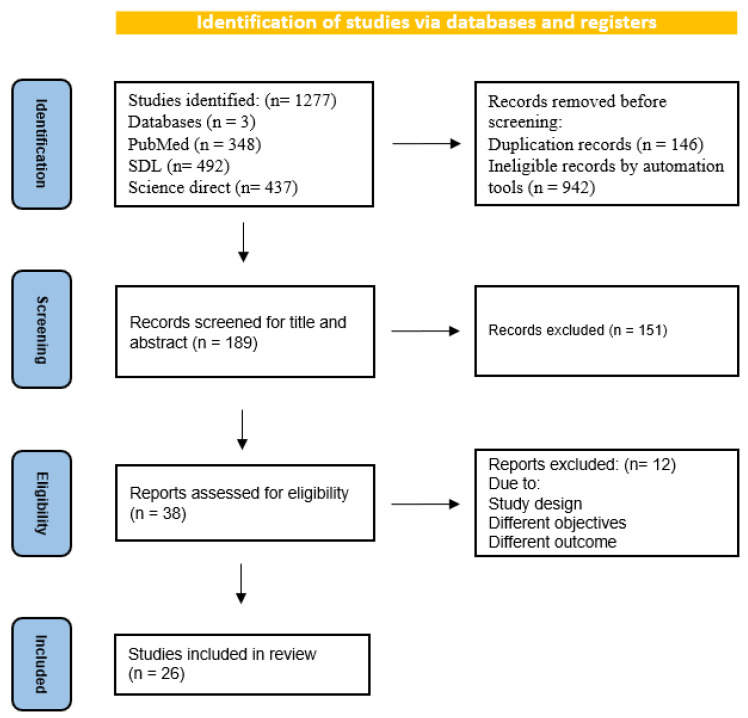
PRISMA flow diagram for search strategy process.

The databases revealed 1277 records of birth defect prevalence and contributing variables in Saudi Arabia through a systematic search based on the PRISMA guidelines from three databases: PubMed (n = 348), SDL (n = 492), and Science Direct (n = 437), as demonstrated in the flow diagram (Figure 3). All identified papers were managed manually, and n= 1088 articles were excluded for duplication (n = 146) or ineligibility by automated tools (n = 942). The titles and abstracts were screened (n = 189), and accordingly, n = 151were excluded (n = 38). Full-text papers were extracted for a more comprehensive evaluation. Twenty-six papers were included in the systematic review and n = 12 were excluded since they are not meeting the inclusion criteria. Seven studies out of the eligible studies were identified to determine the overall prevalence of birth defects [20,23,26,33,38,39,41]. Eighteen additional studies were added for the prevalence of the subtypes of birth defects [21,24,25,27,28,29,30,31,32,34,35,36,37,40,42,43,44,45], and one case report was added for the associated one birth defect [45].

### 3.2. Characteristics of Included Studies

The main features of the included studies that were a part of this systematic review are listed in Table S2. Retrospective study designs were used for 14 (53.8%) of the studies, compared to seven (27%) prospective designs, and there was one case report. These studies were conducted between 1989 and 2020. Eleven studies (42.3%) were conducted in Riyadh [20,23,30,32,33,34,37,38,39,40,44], three (11.5%) in Jeddah [31,35,38], and three(11.5%) in Madinah [21,24,42]. The sample size, excluding the one case report, ranged from 42 in one study to 45,682 in a prospective, cross-sectional, community-based study, including the 13 administrative regions of Saudi Arabia [25,41].

### 3.3. Prevalence of Birth Defects

As shown in Table S2, the highest overall prevalence of birth defects was 46.5 per 1000 live births in a prospective study that included 30,632 babies; this rate was compared to a lower prevalence of 8.6 per 1000 in a retrospective study conducted in Al Ahsa that included 37,168 live births [30,43]. When considering the study period, four prevalence studies (two for Riyadh, one for Al-Ahsa, and one for Al-Khobar) could be included in the prevalence estimates [20,30,36,43]. These four studies reported birth defects from 1992 to 2013, with prevalence rates of 41.5, 46.5, 8.6, and 17 per 1000 live births, respectively. Although another comprehensive study of 13 administrative regions in Saudi Arabia estimated the prevalence of birth defects to be 16.9/1000 in all Saudi regions, it included an age range extending to 19 years old, making it incomparable to other studies [25].

### 3.4. Pattern of Birth Defects

**Figure 4:**
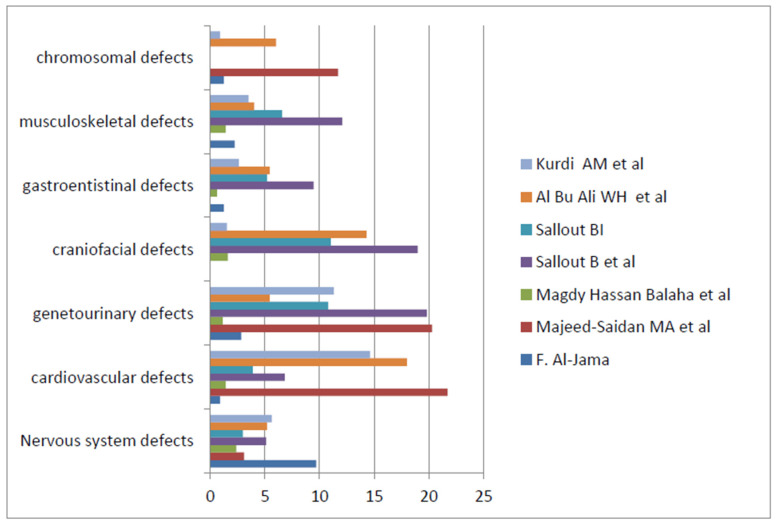
The pattern of birth defects in the main general studies showing the prevalence per 1000 live births [20,23,30,36,42,43,44].

Seven studies assessed the overall birth defects: four in Riyadh, two in Al Ahsa, and one in Al-Khobar. As shown in Figure 4, the most prevalent birth defects were cardiovascular, genitourinary, and craniofacial [20,23,30,36,42,43,44].

Studies involving specific subgroups of birth defects have also been conducted. Five studies reported cleft lip and palate in particular [22,32,33,34,35] and non-syndromic cleft lip and palate (CLP) in affected children. Three studies involved cardiac birth defects and showed the predominance of acyanotic CHDs, namely ventricular septal defects (VSDs) and atrial septal defects (ASDs) [21,26,41]. Generally, congenital heart disease frequently shows a higher prevalence than other birth defects [20,23,30,43,44].Genitourinary defects appeared to have a high prevalence in four studies [20,23,30,44], with one study conducted in Riyadh showing a prevalence of 19.8/1000 live births and an antenatal prevalence of 21.3/1000 [30]. Additionally, another study enrolled 81 children with ambiguous genitalia and concluded that congenital adrenal hyperplasia was the most common cause of this defect [40]. The incidence rate of digestive system defects was 1.3/1000, with imperforate anus and trachea–esophageal fistula/atresia constituting a higher percentage of birth defects [27].Nervous system defects contributed to birth defects in nine studies [20,23,24,30,36,37,39,43,44].Neural tube defects (NTDs) showed percentages ranging between 4.6% to 10.6% [20,23] and a prevalence of 6.1/1000 in one study [30], whereas hydrocephalus showed a prevalence of 1.6/1000 live births [24].Chromosomal abnormalities included Down syndrome (6.6/10,000) as the most typical birth defect [25,29,36,44].

### 3.5. Risk Factors Associated with Birth Defects

Twelve of the included studies [20,22,23,24,25,29,30,31,39,42,43,44] reported relationships between consanguinity and birth abnormalities, which are highly prevalent in the Saudi population. Other risk factors, such as maternal folic acid supplementation, family history of birth defects or genetic abnormalities, and maternal co-morbidities, were reported in several studies [20,24,26,27,36,39,40,43,45]. Male sex was associated with birth defects in two studies [34,35]; however, female sex was a risk factor in one study [28]. Table S3 shows the risk factors in the five studies for each risk’s related odds ratio.

## 4. Discussion

In this systematic review of observational studies, we noted that the prevalence of birth defects in Saudi Arabia ranged from 8.6/1000 to 46.45/1000 [30,43]. However, three extensive studies conducted in Riyadh showed higher prevalence rates of 41.1, 41.2 and 46.4/1000 live births [20,30,44], which were higher than the international prevalence [7,8,9]. This higher prevalence might be attributed to many factors, such as the hospital-based study design used in most studies included in this review. In addition, the enrollment of the referred patients from other areas to Riyadh hospitals, which are specialized tertiary hospitals in the capital of Saudi Arabia, could potentially have increased the prevalence, resulting in inaccurate estimates. Additionally, the high rate of consanguinity in Saudi society, which is a direct and known cause of birth defects, increases the prevalence of certain types of congenital defects compared to international data [46].In the Al Khobar study, the prevalence was 17/1000 live births, which is relatively lower than the global prevalence of birth defects [36]. This result can be compared to that of a comprehensive study of 13 administrative regions in the Kingdom of Saudi Arabia, in which the prevalence of birth defects was estimated to be 16.9/1000 in all regions of the kingdom [25]. However, the age range in the subsequent study was broad, up to 19 years old, which could have influenced the prevalence, resulting in a lower rate because most fatal birth defects could not be accounted for. Moreover, a later study focused on calculating selective patterns of birth defects.

According to WHO estimates, approximately 94% of severe birth defects occur in low- and middle-income countries and are indirectly influenced by the socioeconomic status of these countries [3]. However, the included studies were performed in specific cities of Saudi Arabia and did not provide a conclusive reflection of the Saudi community. Therefore, an epidemiological survey encompassing the whole of Saudi society and considering other variables, such as dietary practices and social, cultural, and other environmental factors, should be employed to determine the actual prevalence of birth abnormalities in any community.

Our study showed that the most frequent birth defects in Saudi Arabia were cardiovascular, genitourinary, and craniofacial defects. In previous studies, the prevalence of cardiovascular birth defects showed a tendency toward acyanotic heart disease, primarily ventricular septal defects [21,26]. Genitourinary defects also showed a higher prevalence that reached 21/1000 in a more extensive study with a predominance of hydronephrosis, which was seen as an isolated birth defect in most infants [30]. Regarding craniofacial anomalies, the most prevalent defect was cleft lip and palate, with a predominance of the non-syndromic type that was significantly correlated with the male [33,34,35]. This distribution pattern of our studies contrasts those in an Arab context, such as an Egyptian study that reported the musculoskeletal system, followed by the central nervous system, as the most common site of birth defects [47]. Another study in Iraq reported that the most common birth defects were in the central nervous system, followed by the musculoskeletal and gastrointestinal systems [5]. Additionally, international data have shown that musculoskeletal and nervous system and cardiovascular birth defects are the most prevalent worldwide [8,9,48,49,50].These discrepancies could be attributed to regional differences in risk factors for particular birth defects.

In this review, the associations of consanguinity with birth defects were reported in 12 studies; however, four extensive studies calculated odds ratios between 1.5 and 3.3, as shown in Table S3 [20,42,43,44]. The Saudi community is well known in the Arab context to have a high prevalence of consanguine marriage that reaches up to 50%, with an average inbreeding coefficient of 0.02265, which is higher than that of many other communities Socio-demographic factors, such as education and regional ancestry, were associated with a propensity for inbreeding [16].

In five studies, we found that maternal factors such as age > 40 years old, obesity with BMI > 30 and maternal diabetes mellitus were significantly associated with birth defects among neonates, with higher odds ratio of 2.1, 7.8 and 2.7, respectively, as shown in Table S3 [20,38,39]. It is well established that advanced maternal age is linked to a significant decrease in the number of healthy, high-quality oocytes produced, increasing the risk of premature births, infertility and congenital birth defects, such as chromosomal abnormalities [51]. Additionally, many studies have reported significant associations of maternal obesity with congenital heart defects, neural tube defects and genitourinary defects [52,53,54]. Diabetes mellitus is a well-established direct cause of birth defects through mechanisms that affect the growing embryo, known as diabetic embryopathy [55].

Many other risk factors showed significant associations with birth defects, such as a family history of birth defects, folic acid supplementation during pregnancy and socioeconomic status; mothers with low socioeconomic status and a positive family history of birth defects were 2.3 times more likely to have babies with birth defects. According to one study, 65–75% of birth defects are believed to have multi-factorial and probable origins. The most common genetic causes are chromosomal abnormalities (5%) and single-gene diseases (15–20%), which are strongly related to a family history of birth defects. Environmental exposures, such as uterine abnormalities, maternal illnesses, drug misuse, infections, medications, radiation, hyperthermia and chemical exposure, caused the remaining 10% of birth abnormalities [10,11,56].

This review provides data on the prevalence and pattern of birth defects in Saudi Arabia. We identified an appropriate number of studies (26 articles) from different regions of Saudi Arabia that showed variable prevalence, patterns and risk factors, reflecting the context of this country compared with global data. However, certain limitations of the current review should be considered before extrapolating. First, most of the included studies were retrospective studies with numerous expected uncontrolled biases in the data collection and enrollment or poor record keeping. Second, abortions and stillbirths were excluded in some studies; subsequently, this omission might have reduced the magnitude of the prevalence by lowering the number of diagnosed birth defect cases, resulting in a discrepancy in prevalence between the studies. Third, the lack of genetic maps to track the genetic problems in particular cases may contribute to the lower prevalence of chromosomal or genetic problems compared to other patterns of birth defects. Finally, the various patterns and proportions of consanguinity reduce the degree of national generalization of the results but contribute a nugget of knowledge to what is already known.

## 5. Conclusions

Birth defects were discovered to be significantly more prevalent in Saudi Arabia than expected based on data from other countries. This high prevalence may be partially explained by the high rate of consanguinity, the hospital-based study design based on survey methodology or studies confined to certain hospitals reflecting the limitations of Saudi society. Further comprehensive, multicenter research in all regions of Saudi Arabia to describe the prevalence is recommended. In addition, it is necessary to establish a Saudi registry for birth defects and a database for the regional distributions of fetal malformations.

## Data Availability

The data underlying this article are available in the article and in its online Supplementary Material.

## Supplementary Materials

The following supporting information can be downloaded at: https://www.mdpi.com/article/10.3390/pediatric15030040/s1, Table S1. PRISMA 2020 Checklist; Table S2. The study characteristics; Table S3. Risk factors associated with birth defects.

## References

[1] Mannan M., Afroze S., Dey S.K., Moni S.C., Shabuj M.K.H., Jahan I., Sultana S.N., Shahidullah M. (2019). Birth Defect and Its Impact in Neonatal Health: A Review. Bangladesh J. Child Health.

[2] Bale J.R., Stoll B.J., Lucas A.O., Institute of Medicine (US) Committee on Improving Birth Outcomes (2003). Improving Birth Outcomes: Meeting the Challenge in the Developing World.

[3] World Health Organization (2023). Congenital Disorders. https://www.who.int/news-room/fact-sheets/detail/birth-defects.

[4] Martínez-Frías M.L. (1994). Developmental field defects and associations: Epidemiological evidence of their relationship. Am. J. Med. Genet..

[5] Ameen S.K., Alalaf S.K., Shabila N.P. (2018). Pattern of congenital anomalies at birth and their correlations with maternal characteristics in the maternity teaching hospital, Erbil city, Iraq. BMC Pregnancy Childbirth.

[6] Zarocostas J. (2006). Serious birth defects kill at least three million children a year. BMJ.

[7] Egbe A.C. (2015). Birth Defects in the Newborn Population: Race and Ethnicity. Pediatr. Neonatol..

[8] Dolk H., Loane M., Garne E. (2010). The prevalence of congenital anomalies in Europe. Adv. Exp. Med. Biol..

[9] Adane F., Afework M., Seyoum G., Gebrie A. (2020). Prevalence and associated factors of birth defects among newborns in sub-Saharan African countries: A systematic review and meta-analysis. Pan Afr. Med. J..

[10] Harris B.S., Bishop K.C., Kemeny H.R., Walker J.S., Rhee E., Kuller J.A. (2017). Risk Factors for Birth Defects. Obs. Gynecol. Surv..

[11] Savitz D.A., Schwingl P.J., Keels M.A. (1991). Influence of paternal age, smoking, and alcohol consumption on congenital anomalies. Teratology.

[12] Wilson R.D., Davies G., Désilets V., Wilson R.D., Davies G., Désilets V., Reid G.J., Summers A., Wyatt P., Young D. (2003). The use of folic acid for the prevention of neural tube defects and other congenital anomalies. J. Obs. Gynaecol. Can..

[13] Gill S.K., Broussard C., Devine O., Green R.F., Rasmussen S.A., Reefhuis J., National Birth Defects Prevention Study (2012). Association between maternal age and birth defects of unknown etiology: United States, 1997–2007. Birth Defects Res. Part A.

[14] Romitti P.A. (2007). Utility of family history reports of major birth defects as a public health strategy. Pediatrics.

[15] Almuneef M., Saleheen H., Albuhairan F., Al-Eissa M., Al Muntaser M., Al Alem H., Baylon B. (2021). A Child mortality in Saudi Arabia: Time for action at all levels. Int. J. Pediatr. Adolesc. Med..

[16] Al Husain M., Al Bunyan M. (1997). Consanguineous marriages in a Saudi population and the effect of inbreeding on prenatal and postnatal mortality. Ann. Trop. Paediatr..

[17] Page M.J., McKenzie J.E., Bossuyt P.M., Boutron I., Hoffmann T.C., Mulrow C.D., Moher D. (2021). The PRISMA 2020 statement: An updated guideline for reporting systematic reviews. J. Clin. Epidemiol..

[18] World Health Organization (2004). International Statistical Classification of Diseases and Related Health Problems.

[19] Checklist for Systematic Reviews and Research Syntheses—Jbi. https://jbi.global/sites/default/files/202008/Checklist_for_Systematic_Reviews_and_Research_Syntheses.pdf.

[20] Taura M.G., Alshahrani A.M., Alqahtani D.O. (2021). Prevalence of congenital heart disease among patients with Down syndrome in Southwestern Saudi Arabia. Ann. Afr. Med..

[21] Khoshhal S.Q., Albasri A.M., Morsy M.M.F., Alnajjar A.A. (2020). The trends and patterns of congenital heart diseases at Madinah Cardiac Center, Madinah, Saudi Arabia. Saudi Med. J..

[22] Alyami B., Ali-Hassan M., Braimah R., Al-Mahri M., Alyami F., Alharieth S. (2020). Prevalence and Clinical Case Series of Syndromic and Nonsyndromic Cleft Lip and Palate in a Saudi Arabian Neonatal Population. Cleft Palate-Craniofacial J..

[23] Sallout B.I., Al-Hoshan M.S., Attyyaa R.A., Al Suleimat A.A. (2008). Antenatal diagnosis, prevalence and outcome of major congenital anomalies in Saudi Arabia: A hospital-based study. Ann. Saudi Med..

[24] Murshid W.R., Jarallah J.S., Dad M.I. (2000). Epidemiology of infantile hydrocephalus in Saudi Arabia: Birth prevalence and associated factors. Pediatr. Neurosurg..

[25] AlSalloum A., El Mouzan M.I., AlHerbish A., AlOmer A., Qurashi M. (2015). Prevalence of selected congenital anomalies in Saudi children: A community-based study. Ann. Saudi Med..

[26] Alabdulgader A.A. (2001). Congenital heart disease in 740 subjects: Epidemiological aspects. Ann. Trop. Paediatr..

[27] Asindi A.A., Al-Daama S.A., Zayed M.S., Fatinni Y.A. (2002). Congenital malformation of the gastrointestinal tract in Aseer region, Saudi Arabia. Saudi Med. J..

[28] Assiry A.A., Khan S.D., Al-Shubrmi H.R., Al-Shammary D.H., Al-Fahhad H.M., Al-Shammary A.F., Al-Shammari A.F. (2020). Head and Neck Congenital Anomalies in Neonate Hospitals in Hail, Saudi Arabia. Int. J. Clin. Pediatr. Dent..

[29] El-Attar L.M., Bahashwan A.A., Bakhsh A.D., Moshrif Y.M. (2021). The prevalence and patterns of chromosome abnormalities in newborns with major congenital anomalies: A retrospective study from Saudi Arabia. Intractable Rare Dis. Res..

[30] Sallout B., Obedat N., Shakeel F., Mansoor A., Walker M., Al-Badr A. (2015). Prevalence of major congenital anomalies at King Fahad Medical City in Saudi Arabia: A tertiary care centre-based study. Ann. Saudi Med..

[31] Bondagji N.S. (2014). Antenatal diagnosis, prevalence and outcome of congenital anomalies of the kidney and urinary tract in Saudi Arabia. Urol. Ann..

[32] AlHayyan W.A., Al Hayek S., AlOtabi S.S., AlGhanim S.A. (2021). Birth prevalence of orofacial cleft in a tertiary hospital in Riyadh, Saudi Arabia: A retrospective audit. Saudi Dent. J..

[33] AlHammad Z., Suliman I., Alotaibi S., Alnofaie H., Alsaadi W., Alhusseini S., Aldakheel G., Alsubaie N. (2021). The prevalence of non-syndromic orofacial clefts and associated congenital heart diseases of a tertiary hospital in Riyadh, Saudi Arabia. Saudi Dent. J..

[34] Hadadi A.I., Al Wohaibi D., Almtrok N., Aljahdali N., AlMeshal O., Badri M. (2017). Congenital anomalies associated with syndromic and non-syndromic cleft lip and palate. JPRAS Open.

[35] Moshref S.S., Jamal Y.S., Fakiha M., Awan B.A., Alsiny F., Alzhrani F., Ammar H., Bamashmos A., Baamer A. (2017). Non-Syndromic Orofacial Cleft Malformations in Jeddah, Saudi Arabia. J. King Abdulaziz Univ. Med. Sci..

[36] Al-Jama F. (2001). Congenital malformations in newborns in a teaching hospital in eastern Saudi Arabia. J. Obstet. Gynaecol..

[37] Alorainy I.A. (2006). Pattern of congenital brain malformations at a referral hospital in saudi arabia: An MRI study. Ann. Saudi Med..

[38] AlShail E., De Vol E., Yassen A., Elgamal E.A. (2014). Epidemiology of neural tube defects in Saudi Arabia. Saudi Med. J..

[39] Aziza A., Kandasamy R., Shazia S. (2011). Pattern of craniofacial anomalies seen in a tertiary care hospital in Saudi Arabia. Ann. Saudi Med..

[40] Al-Jurayyan N.A. (2011). Ambiguous genitalia: Two decades of experience. Ann. Saudi Med..

[41] Kurdi A.M., Majeed-Saidan M.A., Al Rakaf M.S., AlHashem A.M., Botto L.D., Baaqeel H.S., Ammari A.N. (2019). Congenital anomalies and associated risk factors in a Saudi population: A cohort study from pregnancy to age 2 years. BMJ Open.

[42] Ali W.A.B., Balaha M.H., Al Moghannum M.S., Hashim I. (2011). Risk factors and birth prevalence of birth defects and inborn errors of metabolism in Al Ahsa, Saudi Arabia. Pan Afr. Med. J..

[43] Balaha M.H., Al Bu Ali W.H., Al Aswad L.H., Al Moghannum M.S. (2012). Maternal obesity predict isolated birth defects in live births in Eastern Province of Saudi Arabia. J. Matern. Fetal Neonatal Med..

[44] Majeed-Saidan M.A., Ammari A.N., AlHashem A.M., Al Rakaf M.S., Shoukri M.M., Garne E., Kurdi A.M. (2015). Effect of consanguinity on birth defects in Saudi women: Results from a nested case-control study. Birth Defects Res. Part A.

[45] Kamal N.M., Alzeky A.M., Omair M.R., Attar R.A., Alotaibi A.M., Safar A., Abosabie S.A. (2022). First report of *SYNE1* arthrogryposis multiplex congenita from Saudi Arabia with a novel mutation: A case report. Ital. J. Pediatr..

[46] Stoll C., Alembik Y., Roth M.P., Dott B. (1999). Parental consanguinity as a cause for increased incidence of births defects in a study of 238,942 consecutive births. Ann. Genet..

[47] El Koumi M.A., Al Banna E.A., Lebda I. (2013). Pattern of congenital anomalies in newborn: A hospital-based study. Pediatr. Rep..

[48] Sarmah S., Muralidharan P., Marrs J.A. (2016). Common congenital anomalies: Environmental causes and prevention with folic acid-containing multivitamins. Birth Defects Res. C Embryo Today.

[49] Kumar J., Saini S.S., Sundaram V., Mukhopadhyay K., Dutta S., Kakkar N., Kumar P. (2021). Prevalence & spectrum of congenital anomalies at a tertiary care centre in north India over 20 years (1998–2017). Indian J. Med. Res..

[50] Mai C.T., Isenburg J.L., Canfield M.A., Meyer R.E., Correa A., Alverson C.J., National Birth Defects Prevention Network (2019). National population-based estimates for major birth defects, 2010–2014. Birth Defects Res..

[51] Mikwar M., MacFarlane A.J., Marchetti F. (2020). Mechanisms of oocyte aneuploidy associated with advanced maternal age. Mutat. Res./Rev. Mutat. Res..

[52] Zhu Y., Chen Y., Feng Y., Yu D., Mo X. (2018). Association between maternal body mass index and congenital heart defects in infants: A meta-analysis. Congenit. Heart Dis..

[53] Honein M.A., Moore C.A., Watkins M.L. (2003). Subfertility and prepregnancy overweight/obesity: Possible interaction between these risk factors in the etiology of congenital renal anomalies. Birth Defects Res. A.

[54] Stothard K.J., Tennant P.W., Bell R., Rankin J. (2009). Maternal overweight and obesity and the risk of congenital anomalies: A systematic review and meta-analysis. JAMA.

[55] Gajagowni S., Nair P., Bapat A.C., Vachharajani A.J. (2022). Diabetic Embryopathies. Neoreviews.

[56] Brent R.L. (1999). Utilization of developmental basic science principles in the evaluation of reproductive risks from pre- and postconception environmental radiation exposures. Teratology.

