# Supplementary material for: Prevalence and Pattern of Birth Defects in Saudi Arabia: A Systematic Review of Observational Studies
Source: Pediatr Rep. 2023 Jul 20;15(3):431–41. doi: 10.3390/pediatric15030040 (PMC10366848; doi:10.3390/pediatric15030040)
Supplement: Supplementary file 1 [file pediatrrep-15-00040-s001.zip › pediatrrep-2408255-supplementary.pdf]

Table S1. PRISMA 2020 Checklist

Section and Topic	Item #	Checklist item	Location where item is reported
**TITLE**			
Title	1	Prevalence and pattern of birth defects in Saudi Arabia: A systematic review of observational studies	P 1
**ABSTRACT**			
Abstract	2	See the PRISMA 2020 for Abstracts checklist.	S2
**INTRODUCTION**			
Rationale	3	In Saudi Arabia, birth defects remain the leading cause of death among children, with high rates of consanguineous marriage and genetic diseases. There is lacking of existing knowledge regarding the overall prevalence of birth defects.	P2
Objectives	4	To assess the prevalence of birth defects in Saudi Arabia and determine the pattern according to the available data	P3
**METHODS**			
Eligibility criteria	5	This review included original observational research that includes cohort, case-control, cross-sectional studies, case reports, and case series In addition, systematic reviews, review articles, non-relevant articles, and studies that did not fulfill the eligibility criteria were excluded.	P3
Information sources	6	Electronic literature searches were conducted, including Pub Med (National Library of Medicine), Science Direct, and the Saudi digital library, for studies published between January 1989and January 2022.	P3
Search strategy	7	The Medical Subjects Heading (MeSH Database) and keywords search for non-MeSH data was conducted. The keywords employed for the search were "Prevalence" OR "Epidemiology" AND "Birth defects" OR "Congenital Abnormalities" OR "Congenital Malformation" OR "Congenital Anomalies" AND "Pattern" OR "types" OR "sub-types" AND "Saudi Arabia" OR "KSA". A manual search for identified references of included studies, relevant reviews, and grey literature was performed to find further relevant studies not found in the database search.	P3
Selection process	8	Two researchers (TT and MH) screened studies and assessed their eligibility for inclusion.	P3
Data collection process	9	Data abstraction was mediated by three researchers (KA, EE, and OA). Subsequently, one researcher (EE) assessed the quality of each study.	P4

Section and Topic	Item #	Checklist item	Location where item is reported
Data items	10	PICO has been used to define inclusion criteria as follows:<br>**Population/ Patients**: Patients (males and females of all ages) diagnosed with birth defects, including cardiac, craniofacial, musculoskeletal, nervous, genitourinary, gastroenterological, and chromosomal defects. Study eligibility criteria: Inclusion Criteria: Patients diagnosed with birth defects, studies that calculate the prevalence of birth defects, studies that calculate risk factors of birth defects, and studies reporting the characteristics of different patterns of birth defects. The exclusion criteria were studies involving non-humans, opinion papers, editorials, previous reviews, studies validating epidemiological methods, or non-accessible articles were excluded.<br>**Intervention/ Exposure:** This review summarizes data on the epidemiology of birth defects, specifically the prevalence.<br>**Control**: Not Applicable.<br>**Outcomes**: The primary outcomes of this systematic review were the prevalence and patterns of birth defects in Saudi Arabia. The number of newborns delivered with birth defects divided by the total number of babies born during the study period who were enrolled in the study multiplied by 1000, was calculated to estimate the prevalence. At the same time, the main group patterns of birth defects were broadly classified according to the International Classification of Diseases coding system into the nervous system, cardiovascular, genitourinary, craniofacial, musculoskeletal, gastrointestinal, and chromosomal defects. In addition, subgroups were considered as some studies reported specific patterns like cleft lip and palate or congenital heart disease alone. Other outcomes, like the risk factors and clinical outcomes also were also considered.	P4
Study risk of bias assessment	11	*Joanna Briggs Institute* (JBI) provides freely available critical assessment tools for systematic reviews, designed to be study-specific, and presented as checklist questions. Critical assessment checklist for observational studies is used to evaluate for quality of the studies, reliability, validity, and relevance to practice. Two JBI checklists were used: one for cohort studies and the other for cross-sectional studies. one researcher (EE) assessed the quality of each study.	P5
Effect measures	12	Not applicable	-
Synthesis methods	13	Not applicable	-
Reporting bias assessment	14	Not applicable	-
Certainty assessment	15	For certainty of evidence, (GRADE) working group graded the evidence used and rated it as high, moderate, low, and very low certainty (GRADE). Subgroup and stratified analyses were performed according to age,	P5

Section and Topic	Item #	Checklist item	Location where item is reported
		sex, and birth defect patterns.	
**RESULTS**			
Study selection	16a	The databases revealed 1277 records of birth defects prevalence and contributing variables in Saudi Arabia through a systematic search based on PRISMA guidelines from three databases; PubMed (n= 348), SDL (n= 492), and Science Direct (n= 437). All identified papers were managed manually, and (n= 1088) of articles were excluded for duplication (n= 146) or ineligibility by automated tools (n= 942). r The titles and abstracts were screened (n= 189), and accordingly, (n= 151) were excluded. (n= 38) full-text papers were extracted for a more comprehensive evaluation. Twenty-six papers were included in the systematic review, and (n= 12) were excluded as they are not fulfilling the inclusion criteria.	P6
	16b	Seven studies out of the eligible studies were identified to determine the overall prevalence of birth defects [20, 23, 26, 33, 38, 39, 41]. Eighteen additional studies were added for the prevalence of the subtypes of birth defects [21, 24, 25, 27–32, 34–37, 40, 42–45], and one case report was added for the associated one birth defect [45].	P6
Study characteristics	17	Retrospective study designs were used for 14 (53.8%) of the studies, compared to seven (27%) prospective designs, and there was one case report. These studies were conducted between 1989 and 2020. Eleven studies (42.3%) were conducted in Riyadh [20,23,30,32–34,37–40,44], three (11.5%) in Jeddah [31,35,38], and three(11.5%) in Madinah [21,24,42]. The sample size, excluding the one case report, ranged from 42 in one study to 45,682 in a prospective, cross-sectional, community-based study, including the 13 administrative re-gions of Saudi Arabia [41,25].	P6
Risk of bias in studies	18	Present assessments of risk of bias for each included study in figure 2 and 3.	Figure 2&3
Results of individual studies	19	**Prevalence of birth defects:**<br>the highest overall prevalence of birth defects was 46.5 per 1000 live births in a prospective study that included 30,632 babies; this rate was compared to a lower prevalence of 8.6 per 1000 in a retrospective study conducted in Al Ahsa that included 37,168 live births [30,43]. When considering the study period, four prevalence studies (two for Riyadh, one for Al-Ahsa, and one for Al-Khobar) could be included in the prevalence estimates [20,30,36,43]. These four studies reported birth defects from 1992 to 2013, with prevalence rates of 41.5, 46.5, 8.6, and 17 per 1000 live births, respectively. Although another compre-hensive study of 13 administrative regions in Saudi Arabia estimated the prevalence of birth defects to be 16.9/1000 in all Saudi regions, it included an age range extending to 19 years old, making it incomparable to other studies [25].<br>**Pattern of birth defects:**<br>Seven studies assessed the overall birth defects: four in Riyadh, two in Al Ahsa, and one in Al-Khobar. As	P6,7,8

Section and Topic	Item #	Checklist item	Location where item is reported
		shown in Figure 4, the most prevalent birth defects were cardiovas-cular, genitourinary, and craniofacial [20,23,30,36,42–44].<br>Studies involving specific subgroups of birth defects have also been conducted. Five studies reported cleft lip and palate in particular [22,32–35] and non-syndromic cleft lip and palate (CLP) in affected children. Three studies involved cardiac birth defects and showed the predominance of acyanotic CHDs, namely ventricular septal defects (VSDs) and atrial septal defects (ASDs) [21,26,41]. Generally, congenital heart disease frequently shows a higher prevalence than other birth defects [20,23,30,43,44].Genitourinary defects appeared to have a high prevalence in four studies [20,23,30,44], with one study conduct-ed in Riyadh showing a prevalence of 19.8/1000 live births and an antenatal prevalence of 21.3/1000 [30]. Additionally, another study enrolled 81 children with ambiguous genitalia and concluded that congenital adrenal hyperplasia was the most common cause of this defect [40]. The incidence rate of digestive system defects was 1.3/1000, with imperforate anus and trachea–esophageal fistula/atresia constituting a higher percentage of birth de-fects [27].Nervous system defects contributed to birth defects in nine studies [20,23,24,30,36,37,39,43,44].Neural tube defects (NTDs) showed percentages ranging be-tween4.6% to 10.6% [20,23] and a prevalence of 6.1/1000 in one study [30], whereas hy-drocephalus showed a prevalence of 1.6/1000 live births [24].Chromosomal abnormalities included Down syndrome (6.6/10,000) as the most typical birth defect [25,29,36,44].<br>**Risk factors associated with birth defects:**<br>Twelve of the included studies [20,22–25,29–31,39,42–44] reported relationships be-tween consanguinity and birth abnormalities, which are highly prevalent in the Saudi population. Other risk factors, such as maternal folic acid supplementation, family history of birth defects or genetic abnormalities, and maternal co-morbidities, were reported in several studies [20,24,26,27,36,39,40,43,45]. Male sex was associated with birth defects in two studies [34,35]; however, female sex was a risk factor in one study [28].	
Results of syntheses	20	risk of bias among contributing studies was concluded in figure 2&3.	Figure 2&3
Reporting biases	21	Not applicable	-
Certainty of evidence	22	For certainty of evidence, (GRADE) working group graded the evidence used and rated it as high to moderate grade	
**DISCUSSION**			
Discussion	23a	The prevalence of birth defects in Saudi Arabia ranged from 8.6/1000 to 46.45/1000. The most frequent birth defects in Saudi Arabia were cardiovascular, genitourinary, and craniofacial defects. In this review, the	P8,9,10

Section and Topic	Item #	Checklist item	Location where item is reported
		associations of consanguinity with birth defects were reported in 12 studies; however, four extensive studies calculated an odds ratio between 1.5 - 3.3. In five studies, we found that maternal factors such as age > 40 years, obesity with BMI > 30, and maternal diabetes mellitus were significantly associated with birth defects among neonates, with higher odds ratio of 2.1, 7.8, and 2.7, respectively. Many other risk factors showed significant association with birth defects, such as family history of birth defects, folic acid supplementation during pregnancy, and socioeconomic status.	
	23b	Certain limitations on the current review should be considered before extrapolating. First, most of the included studies were retrospective studies with numerous expected uncontrolled biases in the data collection, enrolment, or poor record keeping. Second, abortions and stillbirths were excluded in some studies; subsequently, this might reduce the magnitude of the prevalence by lowering the number of diagnosed birth defect cases, giving the prevalence discrepancy between the studies. Third, the lack of genetic maps to track down the genetic problems in particular cases may contribute to the lower prevalence of chromosomal or genetic problems compared to other patterns of birth defects. Finally, the various patterns and proportions of consanguinity reduce the degree of national generalization of results but contribute a nugget of knowledge to what is already known.	P10
	23c	No limitations of the review processes used.	-
	23d	The implications of the results provide data on the prevalence and pattern of birth defects in Saudi Arabia. Further comprehensive multicenter research in all regions of Saudi Arabia to describe the prevalence is recommended. In addition, it is necessary to establish a Saudi registry for birth defects and a database for the regional distributions of fetal malformations.	P11
**OTHER INFORMATION**			
Registration and protocol	24	The International Prospective Registry of Systematic Reviews (PROSPERO) registered this systematic review protocol on February 25, 2023, under the registration number CRD42023398821.	P3
Support	25	Self funded	P11
Competing interests	26	The authors declare that there is no conflict of interest	P11
Availability of data, code and other materials	27	*The data underlying this article are available in the article and in its online supplementary material*	P11

Table S2.The study characteristics

Author	Study design / Year	Region	Sample size/ source of the information	Characteristics of the patients' with birth defects					Estimated prevalence or incidence of the birth defects
				Age	Gender	pattern of birth defect	Intervention used for the diagnosis	Presence of other co-morbidities or risk	
Kurdi AM et al	Prospective cohort with nested casecontrol study 2010-2013	Riyadh	1,179 live birth / hospital based	20 weeks gestational age up to birth	-	CHD 36%<br>renal 27.4%<br>Nervous system 13.6%<br>NTD 4.6%<br>Digestive system 6.3%<br>CLP 3.6%<br>Limb 8.4%	neonatal screening examination	Lack of peri-conception folic acid.<br>consanguinity,<br>high body mass index,<br>advanced maternal age,<br>smoking<br>maternal diabetes	41.2/1 000
Taura MG et al	cross-sectional study 2016-2019	Bisha	42	1 month–15 years	19 males and 23 females	CHD	echocardiography	Down syndrome	81%
Kamal NM et al	Case report 2022	Saudi Arabia	1/ hospital base	6 months live birth baby	-	Arthrogryposis multiplex congenita ventricular septal defect	Molecular genetic analysis Echocardiogram	Genetic mutation in the family	-
Al Bu Ali WH et al	retrospective case control study 2006-2009	Al Ahsa	38,001/ systemic registry sheet	1-7 days live birth babies	-	craniofacial malformations 61<br>cardiac 51<br>external genitalia 42<br>and multiple birth defects 66	Radiological &sonographic evaluation	rural residence<br>consanguinity<br>prematurity	426 (1.14%)

Khoshhal SQ et al	cohort retrospective 2017-2019	Madinah	1,127 patients with CHDs/ hospital database	3 days to less than 18 years	51.8% males 48.2% females	acyanotic CHDs 84.8% cyanotic CHDs 13% complex 2.2%	imaging studies, and laboratory data	-	-
Alyami B et al	Retrospective Cross - sectional 2013-2016	Najran	24 367/ medical records	One day live birth babies	10 males 5 females and 1 undetermined sex	9 non syndromic CLP 7 having syndromic CLP	Clinical examination	consanguinity	16 (0.65 per 1000)
Sallout BI	prospective study 2005-2007	Riyadh	7762 mother and 5379 babies/ hospital-based	12-18 weeks gestational age	-	Genitourinary 84(38.6%) Cranial 62(28.6%) Skeletal 51(23.5%) Abdominal 40(18.4%) Cardiac 30(13.8%) NTD 23(10.6%)	Antenatal ultrasound	consanguinity	Antenatal prevalence (27.96 / 1000) birth prevalence was 34.57 /1000
Murshid WR et al	prospective / cross sectional study 1996-1997	Madinah	16,550/ hospital-based	1- 28 days Live birth babies	15 males 11 females	Hydrocephalus	head circumference	Multiple pregnancies 21 (81%) Consanguinity 19(73%) Positive family history 4 (15.4%)	26 (1.6 per 1,000)
AlSalloum A et al	prospective, cross-sectional, 2004-2005	13 administrative regions of Saudi Arabia	45 682/ household community based visits	all children below the age of 19 years	-	Down syndrome 30 (6.6/10000) congenital deafness 22 (4.8/10000) congenital blindness 6 (1.3/10000)	Physical examination	Maternal age consanguinity	16.9/10000
Alabdulgader AA	Cross sectional study	Hofuf	50,772 (740 CHD)/ hospital-based	Newborn up to 3 years	351 boys and 389 girls	VSD (39.5%) ASD (11.5%) PS (8.9%) PDA (8.6%)	X-ray, electrocardiogram and echocardiogram	Down syndrome (6%)	CHD incidence of 10.7 / 1000

Asindi AA et al	Prospective cross sectional study 1995-2000	Aseer	1386/ hospital-based	newborn	male/female ratio of 1.7:1	Imperforate anus (78, 44.8%), trache-osophageal fistula/atresia (42, 24.1%)<br>Intestinal atresia (37, 21.3%).<br>Hirschsprung's disease (14 ,8%)<br>stenosis(1.7%)	-	Multi-systemic anomalies	12.4% incidence rate of 1.3 per 1000
Assiry AA et al	Cross sectional 2010-2016	Hail	930 with congenital anomalies/ hospital-based records	One day live birth upto 28 days	Male 51.9%<br>Female 48.1%	Head and neck anomalies	-	Female gender	51 (5.5%)
El-Attar LM et al	retrospective descriptive study 2019-2020	Madinah	2,541/ hospital medical records	One day live birth upto 28 days admitted to NICU	Male 49.3%<br>Female 50.7%	Chromosome abnormalities in 59 (39.3%)<br>Down syndrome (39)<br>Trisomy 18 (5)<br>Trisomy 13 (3)<br>Sex chromosome abnormalities (6)	Images Chromosomal analysis	consanguinity rate was 52.7%	150 ((10.7/1,000)<br>Chromosomal abnormalities 59 (4.22/1,000).
Sallout B et al	prospective cross-sectional 2007-2012	Riyadh	30 632/ hospital-based	Median gestational age was 30 weeks of gestation antenatal age upto one day live birth babies	Male 39.7%<br>Female 40.5%	Antenatal prevalence<br>genitourinary 21.28 / 1000<br>cranial 13.55/1000<br>abdominal 8.98/1000<br>Cardiac 8/1000<br>face and neck 7.5/1000<br>NTD 6.1/1000	Ultrasound	Consanguinity	1598<br>Antenatal prevalence<br>52.1 / 1000<br>46.5 / 1000 live births
Bondagji NS	Cross sectional 2001-2010	Jeddah	43,209/ hospital-based medical records	Antenatal mean gestational age of 26 weeks and Postnatal one day babies	Male 66.7%<br>Female 31.2<br>Non differentiated 2.1%	Hydronephrosis<br>Polycystic kidney disease<br>Multi cystic dysplastic kidney<br>Renal agenesis	Ultrasound	consanguinity	3..26/1000
Wasmiya A et al	Retrospective/ cross sectional study 2014-2018	Riyadh	78 cleft lip and palate cases / birth data registry at		Male 50%<br>Female 50%	Syndromic CLP 0.28/1000<br>Non syndromic CLP 1.57/1000	-	-	1.8/1000

			hospital						
Ziyad AlHammad et al	Retrospective cross-sectional 2015-2018	Riyadh	168 cases of clefts/ hospital-based medical records	1 to 17 years	Male 62% Female 38%	Unilateral CLP 34% (44/130) bilateral CLP 22% (28/130) Associated congenital malformations 41% (54/130	-	CHD was the malformation most commonly associated with OFCs (35%)	non syndromic oro-facial clefts 130/168 (77%)
A.I. Hadadi, D et al	Retrospective cross-sectional 2008-2014	Riyadh	196/ hospital-based	3-24 months,	Male 56% Female 44%	51(26%) CL 78 (40%) CP, 67 (34%) with CLP 38 (19%) were syndromic and 158 (81%) were non-syndromic	-	Male gender	-
Magdy Hassan Balaha et al	retrospective case control study 2006-2010	Al Ahsa	37168/ hospital-based	1-7 days live birth babies	-	nervous system 88 facial defects 61 genitourinary 42 cardiac 51 gastrointestinal 23 musculoskeletal 53	X-ray sonography	body mass index > 30 Consanguinity Low socio-economic status	8.6 / 1000
Majeed-Saidan MA et al	prospective cohort study with nested case-control 2010-2013	Riyadh	28,646/ hospital-based	Live and still birth babies	-	Chromosomal 78 Genetic syndromes 60 Isolated NTD 36 Isolated CHD 265 Isolated renal 239 Isolated others 293 Multiple malformations 152	ultrasound scan , echocardiography, fetal magnetic resonance imaging, and karyotyping	consanguinity	1,179 41.1 per 1000
Moshref, S. S et al	Retrospective cross sectional study 2005-2015	Jeddah	528 with clefts/ hospital-based medical records	0- 12 months	Male 58.6% Female 41.4%	CLP 40.15% Isolated CP 35.61% Isolated CL 24.24%.	-	Male gender smoking	-

F. Al-Jama	Retrospective cross sectional 1992-1997	Al-Khobar	14762/ hospital-based medical records	Live birth babies	male/female ratio 1·2:1	CNS 48.8%<br>musculoskeletal 11.2<br>renal defects 9.2%<br>gastrointestinal 4.8%<br>Cardiac 4.4%<br>GIT 6.1%<br>chromosomal defects 6.1%	-	Diabetic women	17.0/1000
Alorainy, I. A	Retrospective cross sectional	Riyadh	808/ hospital-based MRI reports	children younger than 15 years	Male 48.8%<br>Female 51.2%	Congenital cerebral malformation (NTD, cortical migrational abnormalities, and corpus callosum anomalies) 86 (14.8%)	MRI	-	-
AlShail E et al	Retrospective cross sectional 2000-2012	Riyadh & Jeddah	718 NTD/ hospital-based registry	-	Male 42%<br>Female 58%	-	Ultrasound	Inadequate folic acid intake	-
Aziza A et al	Retrospective cross sectional 2002-2009	Riyadh	447 craniofacial anomalies/ hospital-based registry	-	a male-to-female ratio of 1.18:1.	Craniosynostosis 33.3%<br>ear, face, and neck 39.3%<br>eye 6.6%<br>musculoskeletal 16.5%	-	Consanguinity family history	-
Al-Jurayyan NA	Retrospective Cross sectional 1989-2008	Riyadh	e 81 children with ambiguous genitalia/ hospital based medical records	1 day to 8 years	65.4% genetically females	congenital adrenal hyperplasia 96.1%	chromosomal studies	family history	-

ASD :atrial septal defects CHD : congenital heart disease ,CL: Cleft lip, CP: Cleft palate, CLP: Cleft lip and palate, NTD : neural tube defects, PDA : patent ductus arteriosus , PS : pulmonary stenosis ,VSD : ventricular septal defects, NICU: neonatal intensive care unit

Table S3. Risk factors associated with birth defects

Study	Risk factor	OR	95% CI
Kurdi AM et al [21]	Consanguinity	1.52	1.28 - 1.81
	Maternal age, >40 years	2.11	1.35 - 3.30
	Diabetes mellitus	1.98	1.33 - 2.95
	Sibling with anomalies	1.49	1.04 - 2.12
Al Bu Ali WH et al [23]	Consanguinity	1.54	1.24-1.92
	Rural residence	1.29	1.03-1.61
	Prematurity	1.26	1.02-1.57
Sallout B et al [33]	Nationality (Saudi)	2.22	1.02 - 4.76
	Age (16 to 24)	1.56	1.22 - 2.04
	Family History	2.33	1.75 - 3.13
	Number of Anomalies (>1)	1.52	1.22 - 1.89
Magdy Hassan Balaha et al [38]	Low socio-economic status	2.1 - 2.3	1.18–4.33
	Obesity (BMI > 30)	2.7-7.83	1.3 –15.4
	Consanguinity	3.32	1.54–7.17
Majeed-Saidan MA et al [39]	Consanguinity	1.5	1.2 - 1.9
	Folic acid intake	1.4	1.2 - 1.6
	Diabetes mellitus	2.7	1.4 - 5.4
